# Characterization and Transcriptomic Analysis of Antarctic *Planococcus* sp. Mutant with Enhanced Carotenoid Content

**DOI:** 10.4014/jmb.2512.12033

**Published:** 2026-03-26

**Authors:** Hee-Sun Park, Jong-il Choi

**Affiliations:** Department of Biotechnology and Bioengineering, Chonnam National University, Gwangju 61186, Republic of Korea

**Keywords:** *Planococcus* sp., Carotenoid biosynthesis, EMS mutagenesis, Transcriptome analysis

## Abstract

Carotenoids are industrially important antioxidants, and microbial production represents a sustainable and scalable alternative to plant extraction and chemical synthesis. The recently reported complete genome sequence of the Antarctic bacterium *Planococcus* sp. PAMC21323 indicates its promise as a robust microbial chassis for carotenoid biosynthesis. In this study, we systematically investigated the genetic architecture underlying carotenoid biosynthesis in PAMC21323. Random mutagenesis using ethyl methanesulfonate generated pigment-variant strains, from which a carotenoid-overproducing mutant, R07, was selected through colony pigmentation screening and HPLC analysis. Integrated genome mining, homology-based annotation, and comparative RNA-seq analysis of the wild type and R07 were employed to reconstruct the carotenoid biosynthetic pathway and to characterize global transcriptional changes linked to overproduction. Genome-scale annotation newly defined a CrtM–CrtN–CrtP–CrtNc gene cluster, demonstrating that PAMC21323 synthesizes C_30_ rather than C_40_ carotenoids. Comparative transcriptomic profiling further revealed upregulation of upstream MEP pathway genes and concomitant downregulation of competing non-carotenoid terpenoid branches in R07, indicating effective redirection of metabolic flux toward carotenoid biosynthesis. Collectively, these findings establish the molecular framework for C_30_ carotenoid production in PAMC21323 and highlight a carotenoid-enriched mutant strain as a valuable platform for future metabolic engineering and biotechnological applications in extreme environments.

## Introduction

Industrialization and fossil fuel–based activities have led to the accumulation of diverse organic pollutants and heavy metals in soil, marine, and polar environments. At the same time, climate change has intensified fluctuations in temperature, salinity, and pH, expanding conditions that are unfavorable for many organisms. Under these circumstances, microorganisms capable of simultaneously withstanding combined stresses—such as low temperature, high salinity, and toxic compounds—are gaining attention not only for pollutant remediation but also as bioprocess production platforms that can operate reliably under extreme conditions. In particular, carotenoids are natural antioxidant pigments of growing industrial interest in the food, cosmetic, and functional materials applications, making the establishment of robust production systems based on extremotolerant strains an important industrial challenge [1].

Bacteria of the genus *Planococcus* are frequently isolated from physically and chemically harsh environments, including polar regions, hypersaline lakes, and contaminated sediments, and have been reported to possess diverse stress tolerance traits as well as industrially useful features such as pigment production [2-4]. Among them, *Planococcus* sp. PAMC21323 was isolated from King George Island, Antarctica, and is known to grow relatively stably under low-temperature and saline conditions while producing pigments [5]. Accordingly, *Planococcus* sp. PAMC21323 could serve as a promising microbial chassis for pigment production that can be operated even under extreme or contaminated conditions [4, 5].

Meanwhile, carotenoids reported from several *Planococcus* strains have been suggested to belong to the relatively rare C_30_ class, in contrast to the canonical C_40_ carotenoids typically produced by plants and algae [6-8]. C_30_ carotenoids have distinctive structural features and antioxidant functions, conferring high industrial value; however, it remains unclear which carotenoid pathway predominates in *Planococcus* sp. PAMC21323 [9]. Notably, although the genome sequence is available, the published annotation includes enzyme assignments compatible with both C_30_- and C_40_-associated carotenoid pathways, making it difficult to infer the predominant biosynthetic route from genome annotation alone. Therefore, additional studies are required to functionally characterize the carotenoid biosynthetic pathway of *Planococcus* sp. PAMC21323 and to elucidate the biosynthetic flow of the pigments produced by this strain.

From an industrial perspective, the value of a carotenoid-producing strain depends not only on pathway identification but also on improved productivity [1]. Securing higher-producing variants within the same genetic background streamlines process development and informs subsequent metabolic engineering strategies, thereby increasing the feasibility of practical application [10]. For these reasons, it is meaningful—both academically and applied—to obtain *Planococcus* sp. variants with enhanced carotenoid accumulation and to explain their characteristics at the molecular level.

In this study, we aimed to functionally characterize the carotenoid biosynthetic pathway of *Planococcus* sp. PAMC21323. We further sought to obtain a high-pigment-producing mutant suitable for industrial application [1, 10]. To this end, we constructed an EMS-based mutant library, selected mutants with increased carotenoid production, and compared the selected mutant with the wild type to elucidate the predominant pathway and the molecular basis underlying the high-production phenotype. Because genome annotation alone may be insufficient for carotenoid pathway interpretation, we further integrated comparative transcriptomic analysis between the wild type and the mutant to comprehensively interpret changes in the actively operating biosynthetic pathway and associated metabolic networks. Collectively, this work is expected to deepen our understanding of carotenoid biosynthesis in *Planococcus* sp. PAMC21323 and to provide foundational information for establishing a carotenoid production chassis that can function under extreme and polluted environments.

## Materials and Methods

### Bacterial Strain and Culture Conditions

The bacterial strain used in this study was *Planococcus* sp. PAMC21323, originally isolated from King George Island, Antarctica, and whose complete genome sequence was reported [5]. The strain was obtained from the Polar and Alpine Microbial Collection (PAMC, KOPRI, Republic of Korea). Cells were grown in Tryptic Soy Broth (TSB, 30 g/l) supplemented with 5% (w/v) NaCl and 0.5 M D-sorbitol; for solid media, agar was added to a final concentration of 15 g/l (hereafter referred to as TSB–5% NaCl–0.5 M sorbitol medium) [7] .

For maintenance, the strain was cultured on TSB–5% NaCl–0.5 M sorbitol agar plates at 20–25°C. For liquid cultivation, a single colony was inoculated into 5 ml of the same medium and pre-cultured at 30°C with shaking at 250 rpm for 24 h. The pre-culture was then diluted into fresh medium to an initial OD_600_ of 0.6–0.8, and 50 ml aliquots were transferred into 250 ml baffled Erlenmeyer flasks and incubated at 30°C with shaking at 250 rpm. All experiments were performed under these aerobic shaking conditions. Cell growth was monitored by measuring optical density at 600 nm (OD_600_), and samples for growth measurement, carotenoid quantification, and RNA extraction were collected at 9, 14, 19, 24, and 29 h after inoculation. OD_600_ was measured using a Thermo Scientific Evolution One/One Plus UV-Vis spectrophotometer (USA) with a 1-cm pathlength cuvette.

### EMS Mutagenesis and Mutant Isolation

*Planococcus* sp. PAMC21323 was grown in TSB–5% NaCl–0.5 M sorbitol medium at 30°C with shaking at 250 rpm until reaching mid-exponential phase (OD_600_ = 0.4−0.6), and 1 ml of culture was harvested. Cells were pelleted by centrifugation at 13,000 ×*g* for 5 min at 4°C, the supernatant was discarded, and the pellet was resuspended in 5 ml of fresh medium of the same composition. EMS was then added to the suspension to a final concentration of 3% (v/v), and the mixture was incubated at 30°C with shaking at 250 rpm for 1 h to induce mutations; the conditions were adjusted to achieve a final log reduction of approximately 1.5.

After EMS treatment, cells were centrifuged again at 6,000 ×*g* for 5 min and washed twice with either TSB–5% NaCl–0.5 M sorbitol medium or sterile 1 × PBS. Appropriate dilutions were spread onto TSB–5% NaCl–0.5 M sorbitol agar plates and incubated at 30°C for approximately 3 days to allow colony formation. Colonies were inspected under white light, and those showing altered pigment intensity or distinct pigment phenotypes compared with the wild type were selected and purified for further analysis.

### Carotenoid Extraction and HPLC Analysis

For carotenoid extraction, 6 ml of liquid culture was harvested by centrifugation at 13,000 ×*g* for 5 min at room temperature until a compact cell pellet was obtained. The supernatant was discarded, and 1 ml of methanol was added to the pellet to extract intracellular pigments (culture broth: solvent ratio = 6:1, v/v). The suspension was mixed thoroughly and incubated in a heating block at 60°C for 5 min; if the biomass was not completely bleached, heating was extended in 1-min increments until the pellet became nearly colorless. The extract was then centrifuged at 13,000 ×*g* for 10 min, and the supernatant was transferred to 1.5 ml microcentrifuge tubes. The final supernatant was filtered through a 0.25-μm PTFE syringe filter and transferred to amber HPLC vials.

Chromatographic separation of carotenoids was performed on an HPLC system equipped with a multi-wavelength diode-array detector using a ZORBAX SB-C18 column (250 × 4.6 mm; Agilent Technologies Inc., USA) maintained at 40°C. The mobile phase consisted of solvent A (0.1 M Tris–HCl buffer : acetonitrile : methanol = 14:84:2, v/v/v) and solvent B (methanol : ethyl acetate = 68:32, v/v). Before analysis, the system was purged with both solvents at 3 ml/min and equilibrated in 100% solvent A at 1.2 ml/min for 30 min. Carotenoids were eluted at a flow rate of 1.2 ml/min with the following gradient program: 0–12 min, 100% A; 12–18 min, linear gradient from 100% A to 100% B; 18–20 min, 100% B; 20–25 min, 100% A for re-equilibration. The injection volume was 10 ml, and eluted carotenoids were monitored at 450 nm. Peak areas were used to compare relative carotenoid contents among samples. All experiments were conducted in triplicates.

### RNA Extraction and Sequencing

For transcriptome analysis, *Planococcus* sp. PAMC21323 and the EMS-derived mutant R07 were grown in TSB–5% NaCl–0.5 M sorbitol medium at 30°C with shaking at 250 rpm. Cultures were harvested at 14 h and 24 h after inoculation, and RNA sequencing was ultimately performed for wild type 14 h (*n* = 1), wild type 24 h (*n* = 2), R07 14 h (*n* = 2), and R07 24 h (*n* = 2), respectively. After harvesting, cells were collected by centrifugation, and the resulting pellets were immediately frozen and sent to Macrogen Inc. (Republic of Korea) for whole-transcriptome analysis.

Total RNA extraction, rRNA depletion, and library preparation were all performed by Macrogen. Total RNA was extracted from bacterial pellets and used to construct strand-specific RNA-seq libraries with the SMARTer Stranded RNA library preparation kit (Ribo-Zero, Takara, Japan), according to the manufacturer’s protocol. RNA concentration and integrity were assessed by fluorescence-based quantification and an Agilent 2100 Bioanalyzer, and only samples that met the predefined RNA integrity number (RIN) threshold were used for sequencing. The final libraries were checked for size distribution using a Bioanalyzer, quantified by qPCR, and sequenced on an Illumina platform (2 × 150 bp paired-end).

### Transcriptome and Functional Annotation

Raw Illumina RNA-seq reads were assessed for quality with FastQC v0.11.5 and trimmed with fastp v1.0.1 to remove low-quality bases and adapter sequences. Filtered paired-end reads were aligned to the *Planococcus* sp. PAMC21323 reference genome (GenBank accession no. CP009129.1) using Bowtie2 v2.5.4, and the resulting alignments were sorted and indexed with samtools v1.22.1. Gene-level read counts were obtained from the GFF annotation using featureCounts v2.0.0 (Subread package). Differential expression analysis was performed in R 4.4.3 with DESeq2 v1.46.0, defining differentially expressed genes (DEGs) as those with Benjamini–Hochberg adjusted *p* values (p_adj) < 0.05 and |log_2_(fold change)| ≥ 1. “Raw reads” indicates the total number of sequenced paired-end reads, and “Clean reads” indicates reads remaining after quality trimming and adapter removal [11]. Q30 rate represents the fraction of bases with a Phred quality score ≥ 30, and GC content denotes the proportion of guanine and cytosine bases in the clean reads.

For functional annotation, KEGG Orthology (KO) identifiers were assigned using the KEGG Automatic Annotation Server (KAAS). Protein domains were predicted with HMMER v3.3 against the Pfam-A HMM library (HMMER3 format, indexed in May 2025), and Gene Ontology (GO) terms were mapped using pfam2go (version date 2025-09-01). Carotenoid and MEP/terpenoid pathway genes were further examined by amino acid sequence similarity searches using the UniProt BLAST web server (UniProtKB reference proteomes + Swiss-Prot database). GO and KEGG pathway over-representation analyses and GSEA were carried out using clusterProfiler v4.14.0, and terms or pathways with adjusted *p* values or q values < 0.05 were considered significantly enriched [12].

Whole-genome resequencing reads from the wild type and the EMS-derived mutant R07 were aligned to the same reference genome using minimap2 v2.28-r1209. The resulting BAM files were sorted and indexed with samtools v1.22.1, and single-nucleotide variants and small indels were called with bcftools v1.20 from mpileup-based data. Variants located in the carotenoid biosynthetic gene cluster was extracted from VCF files and manually inspected in Integrative Genomics Viewer (IGV, 2.19.6 desktop version) to confirm well-supported calls and exclude low-depth or ambiguous sites.

## Results and Discussion

### Genome-Wide Identification of Putative Carotenoid Biosynthetic Genes

The original genome annotation of *Planococcus* sp. PAMC21323 contained both C_30_- and C_40_-related carotenoid KOs and enzyme names, creating ambiguity regarding the predominant carotenoid branch in this strain. But, the structures of carotenoids in *Planococcus* sp. PAMC21323 were not identified with LC-MS analysis (data not shown). Although molecular weight signals were obtained, they were insufficient for definitive structural identification of the carotenoid compounds. One possible explanation is that the carotenoids produced by this *Planococcus* strain may represent previously unreported or highly modified C_30_ derivatives for which reference spectra are not currently available. To clarify this, we examined the KEGG carotenoid pathway map and performed BLAST searches to the predicted proteome of PAMC21323 using UniProt reviewed proteins as queries. This analysis identified Plano_0604 as a homolog of 4,4’-diaponeurosporene oxygenase (CrtP) from *Staphylococcus aureus*, exhibiting 59.8% identity and 73.7% positives with an E-value = 0.0. Similarly, putative gene of Plano_2717 was identified as a homolog of 4,4’-diapolycopen-4-al dehydrogenase (CrtNc) from *Metabacillus indicus*, showing 53% identity with an E-value = 0.0. The high sequence identities to annotated putative CrtP and CrtNc from other species and the extremely low E-values (E = 0.0) strongly support annotating Plano_0604 and Plano_2717 as the putative *crtP* and *crtNc* genes, respectively.

Using the same C_30_ pathway map as a framework, we next searched for candidates of CrtM, which catalyzes the head-to-head condensation of two farnesyl diphosphate molecules, and CrtN, which introduces sequential desaturations into 4,4?-diapophytoene. Blastp-based amino acid sequence mining with CrtM proteins from various *Staphylococcus* and CrtN proteins from *Bacillus* [13, 14] as queries, identified Plano_2716 as a dehydrosqualene synthase–type CrtM homolog and Plano_2718 as a carotenoid desaturase–type CrtN homolog, respectively. Both candidates showed ~30% sequence identity, alignment lengths of 270–500 amino acids, >90% query coverage, and strong statistical support.

**Fig. 1:**
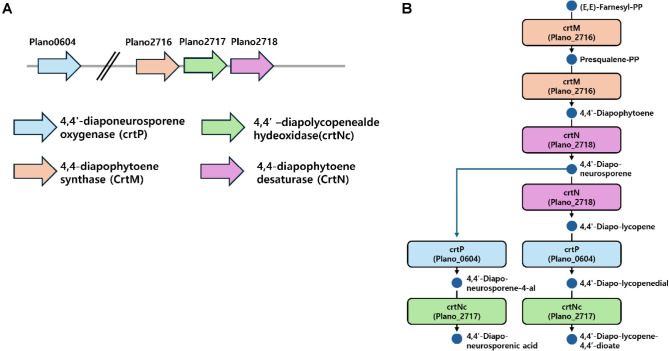
(A) Proposed C_30_ carotenoid biosynthetic genes on chromosome in *Planococcus* sp. PAMC21323 and (B) C_30_ carotenoid biosynthetic pathway, respectively.

Plano_2716, Plano_2717, and Plano_2718 are located adjacently on the chromosome in the order *crtM*–*crtNc*–*crtN*, forming a carotenoid biosynthetic cluster, whereas the *crtP* candidate Plano_0604 is located at a separate chromosomal position. This genomic organization supports the interpretation that PAMC21323 harbors a C_30_ rather than a C_40_ carotenoid pathway, consisting of CrtM–CrtN–CrtP–CrtNc (Fig. 1). Furthermore, there was the absence of lycopene in HPLC profiles of mutants in this study (data not shown). Previous studies also reported that most of *Planococcus* strains were known to produce C_30_ carotenoid as major pigment [15].

### Isolation of EMS-Derived Mutant with Enhanced Carotenoid Production

**Fig. 2:**
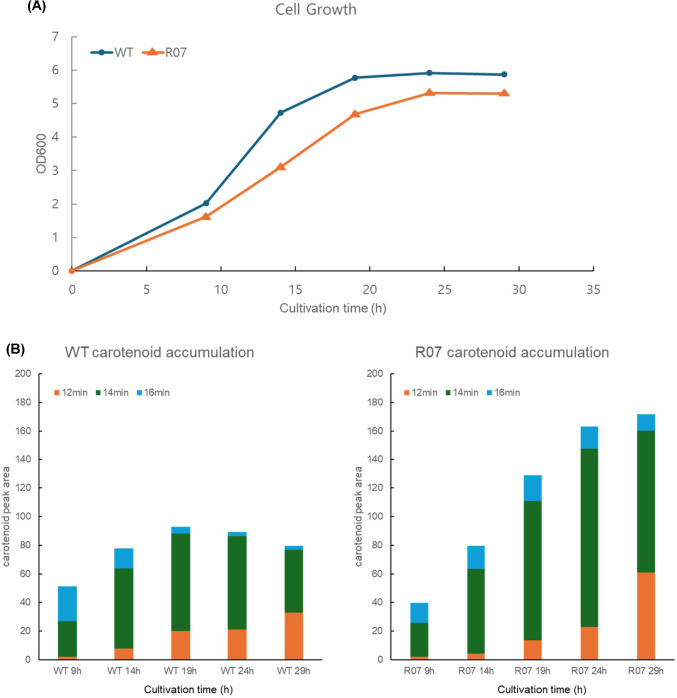
Time-course of (A) growth and (B) carotenoid accumulation in wild type *Planococcus* sp. PAMC21323 and mutant R07, respectively.

EMS mutagenesis of *Planococcus* sp. PAMC21323 produced numerous colonies with altered pigmentation on TSB–5% NaCl–0.5 M sorbitol agar. Representative mutants were selected based on colony coloration under white light, and the mutant R07 exhibited a deeper orange hue compared to the wild type (WT). Carotenoid profiles of the WT and EMS-derived mutant R07 were examined by HPLC analysis. The carotenoids chromatogram of WT exhibited three major peaks with retention times (RTs) of approximately 12, 14, and 16 min, respectively. R07 displayed a similar pattern of peaks at these RTs but with larger peak areas than the WT. The temporal dynamics of growth and carotenoid accumulation were further compared between the WT and the representative high-pigment mutant R07. Cultures were sampled at 9, 14, 19, 24, and 29 h after inoculation, and OD_600_ and carotenoid levels were measured at each time point (Fig. 2). The growth curves showed that R07 consistently reached a little lower OD_600_ values than the WT at all examined time points (Fig. 2A). Despite the reduced cell density, the total carotenoid contents estimated from HPLC peak areas were higher in R07 than in the WT after 14 h, indicating that R07 maintained a high-pigment phenotype over the course of cultivation (Fig. 2B). At 24 h and 29 h points, R07 accumulated approximately two-fold more carotenoids than the WT under the same cultivation conditions. Furthermore, the carotenoid concentration in wild type was the highest at 19 h and decreased, but the concentration in R07 was continuously increased. LC and LC–MS analyses did not reveal clear evidence of changes in carotenoid structure between the wild type and the R07 mutant; rather, the mutant exhibited increased peak intensities corresponding to same retention times.

### RNA-Sequencing and Differential Expression Genes

RNA-sequencing was performed for a total of seven samples of the wild-type strain and the high-pigment mutant R07. During mRNA library construction, one wild-type sample at 14 h was failed, and therefore 7 samples was constructed and sequenced. Each library yielded approximately 2.2 × 10^7^ – 2.4 × 10^7^ raw reads, and 2.1 × 10^7^ – 2.3 × 10^7^ clean reads remained after quality filtering (Table 1). All samples showed Q30 values above 96% and GC contents of 41.0–41.5%, indicating overall high sequencing quality. Filtered reads were mapped to the *Planococcus* sp. PAMC21323 reference genome using Bowtie2, resulting in high mapping rates and uniform coverage (Table 2).

**Fig. 3:**
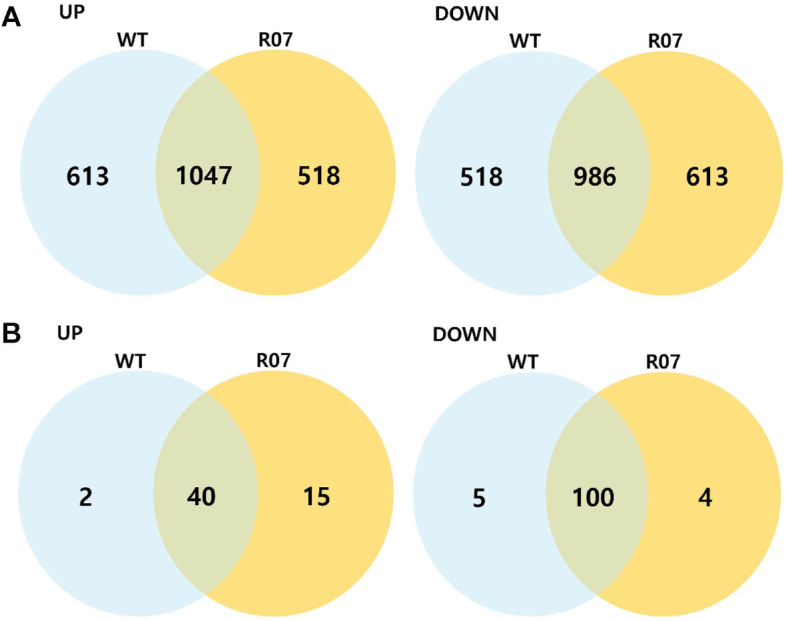
Overlap of differentially expressed genes between wild type *Planococcus* sp. PAMC21323 and mutant R07. (**A**) Venn diagrams summarizing genes with increased (UP, left) or decreased (DOWN, right) expression at 24 h relative to 14 h in each strain without applying statistical thresholds. (**B**) Venn diagrams showing the same comparison restricted to differentially expressed genes satisfying |log_2_(fold change)| ≥ 1 and adjusted *p* value < 0.05.

DESeq2 analysis was first performed without applying statistical filters, considering only the direction of log_2_ fold change in the 24 h versus 14 h comparison. In R07, 1,565 genes showed increased expression at 24 h (518 genes specific to R07 and 1,047 genes common in both strains), whereas 1,599 genes showed decreased expression (613 genes specific to R07 and 986 genes common in both strains). In the WT, 1,660 genes were up-regulated (613 genes specific to WT and 1,047 genes common in both strains) and 1,504 genes were down-regulated (518 genes specific to WT and 986 genes common in both strains) (Fig. 3A). When a more stringent threshold of |log_2_(fold change)| ≥ 1 and adjusted *p* value (p_adj) < 0.05 was applied, R07 had 55 up-regulated (15 genes to R07 and 40 common) and 104 down-regulated genes (4 genes to R07 and 100 common), whereas the WT had 42 up-regulated (2 genes to WT and 40 common) and 105 down-regulated genes (5 genes to WT and 100 common) (Fig. 3B). The list of DEGs was presented in Table S1. Subsequent functional analyses focused on these stringently defined DEG sets.

### Transcriptional and Mutational Analysis of the Carotenoid Biosynthetic Cluster in Mutant R07

**Fig. 4:**
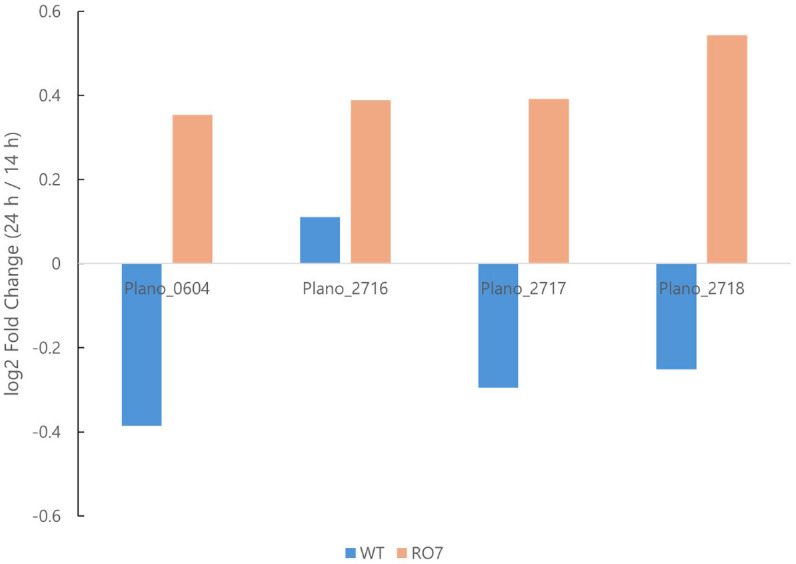
RNA-seq–based expression levels of carotenoid biosynthesis–related genes (putative *crtM*, *crtN*, *crtP*, and *crtNc*) in the WT and R07 harvested at 14 h and 24 h. Time-dependent expression changes (14 h to 24 h) of four carotenoid-related genes (Plano_0604, Plano_2716, Plano_2717, and Plano_2718) in WT (blue) and R07 (orange), shown as log_2_ fold change (24 h to 14 h).

Consistent expression patterns were observed for genes in the carotenoid biosynthetic cluster between strains. The putative *crtM* (Plano_2716), *crtNc* (Plano_2717), *crtN* (Plano_2718), and *crtP* (Plano_0604) genes showed increased expression after 14 h in both strains, but their transcript levels were generally higher in R07 than in the WT at 24 h and tended to remain elevated for a longer period (Fig. 4). Although many of these individual genes did not strictly meet the DEG criteria of |log_2_(fold change)| ≥ 1 and adjusted *p* value < 0.05, the coherent up-shift of the cluster together with the MEP/terpenoid rewiring provides transcriptomic support for the high-pigment C_30_ carotenoid accumulation phenotype observed in R07. The increased expression levels of genes in carotenoid biosynthesis were also confirmed by RT-qPCR (Fig. S1).

To identify mutations accumulated in carotenoid pathway genes of the EMS-derived mutant R07, we performed variant calling of the WT and R07. Filtered reads were reference-based mapped to the *Planococcus* sp. PAMC21323 genome using minimap2, and SNVs and small indels were called using bcftools. Variants located in the carotenoid biosynthetic cluster and in genes of the MEP/terpenoid backbone pathway were extracted and manually inspected in IGV. Several single-nucleotide substitutions were detected, and notably, the *crtNc* candidate Plano_2717 carried an L169F missense mutation resulting from a CTC (Leu) to TTC (Phe) codon change. The mutation was confirmed by sequencing the PCR products of the genes from wild type and R07. This residue corresponds to a position near the β6 region of AldH-family enzymes, which has been reported to participate in gating of the substrate access tunnel [16]. But, the effect of this codon change on the activity of CrtNc was not reported yet.

### MEP/Terpenoid Pathway Gene Expression

**Fig. 5:**
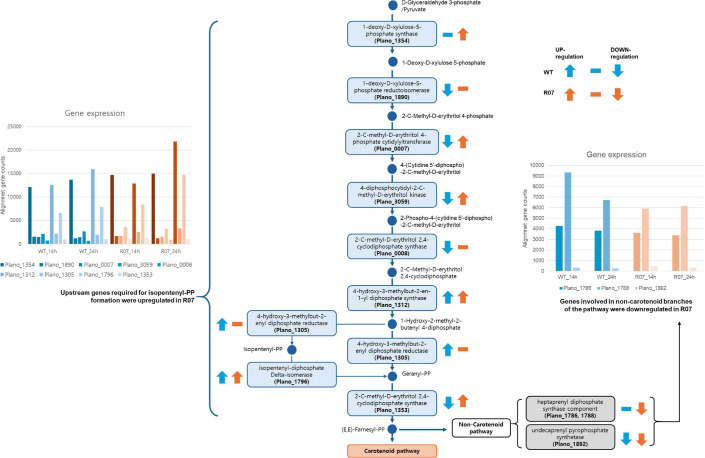
Transcriptome-level rewiring of the MEP and terpenoid backbone pathways in wild type and mutant R07. Expression direction is indicated by arrows (blue for WT, orange for R07). In the accompanying bar plots, the color family denotes the strain (blue: WT; orange: R07), and different shades within each family distinguish individual genes (locus tags).

Because carotenoid biosynthesis relies on precursor supply from the non-mevalonate (MEP) pathway and terpenoid backbone biosynthesis via IPP/DMAPP, genes assigned to these upstream pathways were selected based on KEGG annotations and compared between the WT and R07 using normalized read counts (Fig. 5). In the upper MEP/terpenoid-backbone set, the putative genes, including putative 4-diphosphocytidyl-2-C-methyl-D-erythritol kinase (Plano_3059), putative 2-C-methyl-D-erythritol 2,4-cyclodiphosphate synthase (Plano_0008/Plano_1353), putative 4-hydroxy-3-methylbut-2-en-1-yl diphosphate synthase (Plano_1312), putative 4-hydroxy-3-methylbut-2-enyl diphosphate reductase (Plano_1305), and putative isopentenyl-diphosphate delta-isomerase (Plano_1796), showed higher expression in R07 than in the WT at 24 h (Fig. 4, red-boxed). Building on previous reports that the entry reactions of the MEP pathway—catalyzed by 1-deoxy-D-xylulose-5-phosphate synthase (Plano_1354) and 1-deoxy-D-xylulose-5-phosphate reductoisomerase (Plano_1890)—often constitute a major bottleneck for pathway flux, the higher expression of these genes in R07 relative to the WT already at 14 h suggests that early alleviation of this bottleneck may have increased the supply of upstream isoprenoid precursors [17]. This coordinated increase at the upstream steps suggests an expanded pool of IPP/DMAPP and downstream Farnesyl-PP available for carotenoid biosynthesis.

In contrast, genes mapped to non-carotenoid branches of terpenoid metabolism, including putative heptaprenyl diphosphate synthase components (Plano_1786 and Plano_1788) and putative undecaprenyl pyrophosphate synthase (Plano_1892), displayed lower expression in R07 than in the WT at 24 h (Fig. 4, blue-boxed). Taken together, the higher expression of upstream precursor-supplying genes and the reduced expression of competing non-carotenoid branches are consistent with a shift in terpenoid intermediates toward the carotenoid pathway, in agreement with the elevated carotenoid production observed for R07 relative to the WT (Fig. 2B).

The R07 mutant exhibits a visible growth defect (Fig. 2A). The reduced growth observed in R07 is likely attributable, at least in part, to metabolic burden resulting from increased withdrawal of isoprenoid precursors (IPP/DMAPP) toward carotenoid biosynthesis. In addition, we evaluated the potential structural impact of the identified mutation in mutM through molecular modeling analysis, which indicated no significant alteration in protein structure or substrate affinity (data not shown), suggesting that the mutation itself may not directly impair enzymatic function.

### Other DEGs Potentially Linked to Carotenoid Accumulation

When comparing the 14 h-to-24 h transcriptional shifts between the WT and R07, we identified a set of DEGs showing strain-dependent responses over time. Several of these genes are annotated to functions in central carbon and acetyl-CoA metabolism, β-oxidation, translation, and amino-acid metabolism processes that can influence carotenoid biosynthesis by modulating precursor availability and cellular reducing power (Table 3).

The putative glutamate dehydrogenase gene (Plano_0651) was downregulated in R07 at 24 h. Previous metabolic engineering studies in *Escherichia coli* established that limiting GDH activity enhances the intracellular pool of NADPH, a critical cofactor, thereby boosting the production of terpenoid pigments such as β-carotene and lycopene [18, 19]. Consequently, the suppression of Plano_0651 expression in R07 suggests a potential metabolic rewiring that might conserve NADPH from central amino acid metabolism to fuel the NADPH-intensive carotenoid biosynthetic pathway. Moreover, whole-genome and RNA-seq analyses have shown that reconfiguration of alanine, aspartate, and glutamate metabolism, including downregulation of enzymes such as glutamate-semialdehyde dehydrogenase and prephenate dehydrogenase, is associated with increased carotenoid accumulation in *Phaffia rhodozyma* [20]. Consistent with this, a putative prephenate dehydrogenase gene was also downregulated in R07 relative to the wild type. Together, the transient activation of glutamate dehydrogenase and reduced prephenate dehydrogenase expression suggest broader rewiring of glutamate and aromatic amino-acid metabolism in R07, which may indirectly contribute to its high-pigment phenotype.

Notably, the putative DNA glycosylase gene (Plano_0654) was significantly downregulated exclusively in WT strain at 24 h, a pattern not observed in the R07. Deficiency in DNA glycosylase (*e.g.*, MutM) compromises the base excision repair (BER) pathway, thereby exacerbating intracellular oxidative stress in *Bacillus subtilis* [21]. Consequently, downregulation of Plano_0654 in the WT likely impaired oxidative stress defense, leading to ROS accumulation and non-enzymatic carotenoid degradation (chemical bleaching) [22]. This suggests that compromised stress response contributes to lower pigment levels in the WT, a limitation apparently mitigated in the R07.

Additionally, Plano_0925, annotated as putative a 3-hydroxyacyl-CoA dehydrogenase acting in the third step of β-oxidation, was also upregulated by about threefold in R07 at 24 h relative to the wild type. In torularhodin-producing *Rhodotorula* strains, a ~1.4-fold increase in the 3-hydroxyacyl-CoA dehydrogenase HCD1 is sufficient to raise carotenoid production more than threefold by enhancing acetyl-CoA and NAD(P)H supply [9, 23], suggesting that higher Plano_0925 expression in R07 may play a similar supporting role.

The putative acetyl-CoA ligase gene of Plano_1067 was upregulated by approximately twofold in R07 compared with the wild type. In *Rhodosporidium kratochvilovae*, deletion of the acetyl-CoA synthetase RkACS1 reduces carotenoid content by ~20%, whereas overexpression of RkACS1 or RkACS2 increases carotenoids by 40–60% [24]. By analogy, the elevated expression of Plano_1067 in R07 is likely to increase acetyl-CoA availability and thereby indirectly support its high-pigment phenotype.

In addition, the genes Plano_1910 and Plano_2412, encoding putative 50S ribosomal proteins L19 and L31, respectively, were both upregulated more than twofold in R07 at 24 h. Defects or remodeling of plastid and bacterial ribosomes are known to affect pigment biosynthesis—for example, mutation of plastid ribosomal protein L13 in rice causes strong reductions in both chlorophyll and carotenoids [25], and zinc-responsive alternative ribosomal proteins are required for normal carotenoid production in *Mycobacterium smegmatis* under zinc-limiting stress [26]. Thus, increased expression of Plano_1910 and Plano_2412 in R07 may indirectly favor carotenoid accumulation by adjusting translational capacity or stress adaptation.

The increased transcription levels observed in the mutant strain may indeed result from mutations in promoter or regulatory regions affecting gene expression. Alternatively, mutations in unidentified global or local regulatory genes could indirectly influence transcription of the carotenoid biosynthetic pathway. Because EMS mutagenesis introduces multiple genomic mutations, it is difficult to pinpoint the exact regulatory mechanism at this stage.

## Conclusion

Despite the diversity of carotenoid-producing microorganisms, the biosynthetic pathways and structures of many remain uncharacterized. This study underscores the value of integrating genomic sequencing with mutant phenotypic analysis to elucidate these pathways. Our intensive sequencing analysis provides compelling evidence that *Planococcus* sp. PAMC21323 utilizes a rare C_30_ biosynthetic pathway rather than the canonical C_40_ route. Compared with the canonical C_40_ carotenoids, C_30_ carotenoids may confer several advantages, including efficient antioxidant activity, structural adaptability through functional modifications, and distinct interactions with cellular membranes that could contribute to stress tolerance. These properties may be particularly beneficial under polar environmental conditions, where organisms are exposed to low temperatures, elevated oxidative stress, and increased UV radiation. The isolated R07 mutant serves as a robust model for studying mechanisms of enhanced carotenoid accumulation. Its stable, high-production phenotype suggests significant potential for improving yield and consistency in industrial applications. Furthermore, the transcriptome analysis clarifies the system-level metabolic adjustments in the R07 mutant, providing a foundational framework for future metabolic engineering and commercial strain development. Because construction of recombinant strain will be an important direction for future research, the development of reliable genetic manipulation tools for *Planococcus* will be necessary. Furthermore, transcriptome analysis alone cannot fully explain the mechanisms underlying increased carotenoid accumulation, and additional biochemical analyses, such as enzyme activity measurements or metabolite quantification, would provide more comprehensive insight.

## Supplemental Materials

Supplementary data for this paper are available on-line only at http://jmb.or.kr.



## Figures and Tables

**Table 1:** RNA-seq data quality and read statistics for wild-type *Planococcus* sp. PAMC21323 and mutant R07.

Sample ID	Raw reads	Clean reads	Q30 rate	GC content
R07_14h-2	22,164,670	21,680,086	96.6%	41.2%
R07_14h-3	23,414,364	22,909,006	96.3%	41.1%
R07_24h-10	23,693,794	23,202,390	96.5%	41.1%
R07_24h-12	23,382,710	22,832,408	96.3%	41.0%
WT_14h-5	23,128,550	22,017,822	96.4%	41.1%
WT_24h-11	22,001,384	21,538,182	96.4%	41.3%
WT_24h-6	23,710,950	22,964,314	96.2%	41.5%

**Table 2:** Summary of mapping information coverage.

Samples		Mapped	Unmapped
R07 14h	1	97.28%	2.72%
	2	97.1%	2.9%
R07 24h	1	97.29%	2.71%
	2	96.65%	3.35%
WT 14h	1	97.03%	2.97%
WT 24h	1	94.96%	5.04%
	2	95.63%	4.37%

**Table 3:** List of selected candidate genes showing strain-dependent expression patterns (24 h vs 14 h) in *Planococcus* sp. PAMC21323 wild type and mutant R07.

	Locus_tag	Description
R07 only DOWN	Plano_0651	Glutamate dehydrogenase
WT only DOWN	Plano_0654	DNA glycosylase
R07 only UP	Plano_0925	3-hydroxyacyl-CoA dehydrogenase
	Plano_1067	Acetyl-CoA ligase (synthetase)
	Plano_1910	50S ribosomal protein L19
	Plano_2412	50S ribosomal protein L31
	Plano_0651	Glutamate dehydrogenase
	Plano_0654	DNA glycosylase
