# Supplementary material for: Characterization and Transcriptomic Analysis of Antarctic Planococcus sp. Mutant with Enhanced Carotenoid Content
Source: J Microbiol Biotechnol. 2026 Mar 26;36:e2512033. doi: 10.4014/jmb.2512.12033 (PMC13044350; doi:10.4014/jmb.2512.12033)
Supplement: Supplementary file 1 [file jmb-36-e2512033-supple.pdf]

J. Microbiol. Biotechnol. 2026. 36: e2512033

https://doi.org/10.4014/jmb.2512.12033

Table S1. The list of DEGs between samples at 14 h and at 24 h.

	Locus_tag	Description	p-value
R07 only UP	Plano_0560	fatty acid hydroxylase	4.46.E-02
	Plano_0924	thioredoxin-like protein	7.41.E-05
	Plano_1611	alkaline phosphatase like protein	8.62.E-10
	Plano_1997	Phenylalanyl-tRNA synthetase alpha subunit	3.84.E-05
	Plano_2641	signal peptidase	1.01.E-07
BOTH UP	Plano_0150	Nudix hydrolase	4.95.E-04
	Plano_0163	transposase	2.59.E-03
	Plano_0216	serine protease	2.20.E-06
	Plano_0223	pantothenate kinase	8.34.E-04
	Plano_0254	beta-lactamase domain-containing protein	6.90.E-09
	Plano_0356	Nitrogen regulatory protein	7.37.E-07
	Plano_0365	acetyl-CoA acetyltransferase	1.14.E-03
	Plano_0399	GCN5-related N-acetyltransferase	9.81.E-06
	Plano_0518	transcriptional regulator, MarR family	4.45.E-03
	Plano_0556	putative transposase	4.02.E-02
	Plano_0557	transposase	1.91.E-03
	Plano_0660	AMP-dependent synthetase and ligase	1.51.E-07
	Plano_0832	GTPase	8.05.E-03
	Plano_0901	Nudix hydrolase	2.49.E-05
	Plano_1289	cytochrome c class I	1.47.E-03
	Plano_1557	Molybdenum cofactor biosynthesis protein MoaE	5.53.E-07
	Plano_2171	homoserine kinase	5.31.E-08
	Plano_2172	threonine synthase	5.92.E-08
	Plano_2256	mannose-6-phosphate isomerase	2.97.E-03
	Plano_2257	mannose-6-phosphate isomerase	4.46.E-05
	Plano_2542	transcriptional regulator, BadM/Rrf2 family	9.41.E-05
	Plano_2597	two-component response regulator	1.99.E-03
	Plano_2889	organic hydroperoxide resistance protein	2.82.E-02

	Plano_3030	pyridoxal biosynthesis protein	1.19.E-02
R07 only DOWN	Plano_0651	glutamate dehydrogenase	4.64.E-09
	Plano_0683	NAD-dependent epimerase/dehydratase	3.93.E-12
	Plano_1541	putative methyltransferase	1.91.E-03
WT only DOWN	Plano_0654	DNA glycosylase	1.76.E-04
BOTH DOWN	Plano_0222	general stress protein	3.09.E-06
	Plano_0310	Magnesium and cobalt transport protein CorA	6.93.E-04
	Plano_0350	glycine/betaine ABC transporter permease	1.67.E-08
	Plano_0375	intracellular protease, PfpI family protein	5.92.E-04
	Plano_0427	UDP-N-acetylmuramoylalanyl-D-glutamate--2,6-diaminopimelate ligase	7.39.E-05
	Plano_0443	phosphoserine phosphatase	8.83.E-09
	Plano_0561	PQQ-dependent oxidoreductase, gdhB family	2.09.E-05
	Plano_0598	anti-sigma-factor antagonist	1.87.E-08
	Plano_0599	putative flavoenzyme	7.55.E-14
	Plano_0600	NAD dependent epimerase/dehydratase	7.06.E-08
	Plano_0641	phage-shock protein	1.13.E-05
	Plano_0650	S-layer domain-containing protein	1.78.E-03
	Plano_0664	short chain dehydrogenase	1.66.E-03
	Plano_0669	sulfatase	5.50.E-23
	Plano_0770	succinyl-diaminopimelate desuccinylase	2.17.E-26
	Plano_0784	methyltransferase	1.30.E-09
	Plano_0990	2-succinyl-5-enolpyruvyl-6-hydroxy-3-cyclohexene-1-carboxylate synthase	1.16.E-05
	Plano_0991	2-succinyl-6-hydroxy-2,4-cyclohexadiene-1-carboxylate synthase	3.26.E-09
	Plano_1032	haloacid dehalogenase family protein	1.04.E-07
	Plano_1033	peroxiredoxin	3.04.E-11
	Plano_1048	tRNA (guanine-N(7)-)-methyltransferase	3.94.E-04
	Plano_1049	putative metal-dependent hydrolase	4.96.E-05
	Plano_1110	malate dehydrogenase	1.32.E-06
	Plano_1356	arginine repressor	7.11.E-06
	Plano_1367	2-oxoisovalerate dehydrogenase subunit alpha	2.16.E-03
	Plano_1399	isochorismatase hydrolase	4.51.E-02

Plano_1438	U61 family carboxypeptidase	4.01.E-02
Plano_1475	FAD-dependent pyridine nucleotide-disulfide oxidoreductase	3.39.E-08
Plano_1713	Magnesium and cobalt transport protein	3.26.E-14
Plano_1847	aluminum resistance protein	1.50.E-06
Plano_1993	glycerophosphoryl diester phosphodiesterase	2.50.E-05
Plano_2024	pyruvate dehydrogenase E1 subunit beta	7.12.E-09
Plano_2090	inorganic polyphosphate/ATP-NAD kinase	1.75.E-03
Plano_2142	3-ketoacyl-ACP reductase	1.97.E-04
Plano_2161	gamma-glutamyl-gamma-aminobutyrate hydrolase	3.00.E-09
Plano_2217	putative UV damage endonuclease	1.72.E-03
Plano_2366	sodium:alanine symporter	3.58.E-16
Plano_2377	Muramoyltetrapeptide carboxypeptidase	1.24.E-03
Plano_2447	spermidine synthase	5.57.E-07
Plano_2503	putative diacylglycerol kinase	2.31.E-07
Plano_2517	membrane protein	1.96.E-14
Plano_2556	putative ribonuclease BN	3.22.E-05
Plano_2568	Zn-dependent hydrolase	7.42.E-15
Plano_2586	glycosyltransferase family protein	1.89.E-09
Plano_2589	superoxide dismutase	1.12.E-08
Plano_2590	short-chain dehydrogenase/reductase SDR	6.09.E-03
Plano_2662	acetyltransferase	1.09.E-09
Plano_2664	putative beta-L-N-acetylhexosaminidase	4.15.E-07
Plano_2665	glycosyl transferase family protein	1.73.E-12
Plano_2898	dioxygenase	1.21.E-04
Plano_2955	cation transporter	3.56.E-12

Locus_tag indicates the gene identifier in the *Planococcus* sp. PAMC21323 genome. R07 only UP, WT only DOWN, and R07 only DOWN denote genes exhibiting strain-specific differential expression patterns at 24 h relative to 14 h. Candidate genes were selected using the criteria of an absolute log2 fold change (|log2FC|) > 1 and *p*-value < 0.05. E-value also represents the statistical significance of the homology search used for functional annotation.

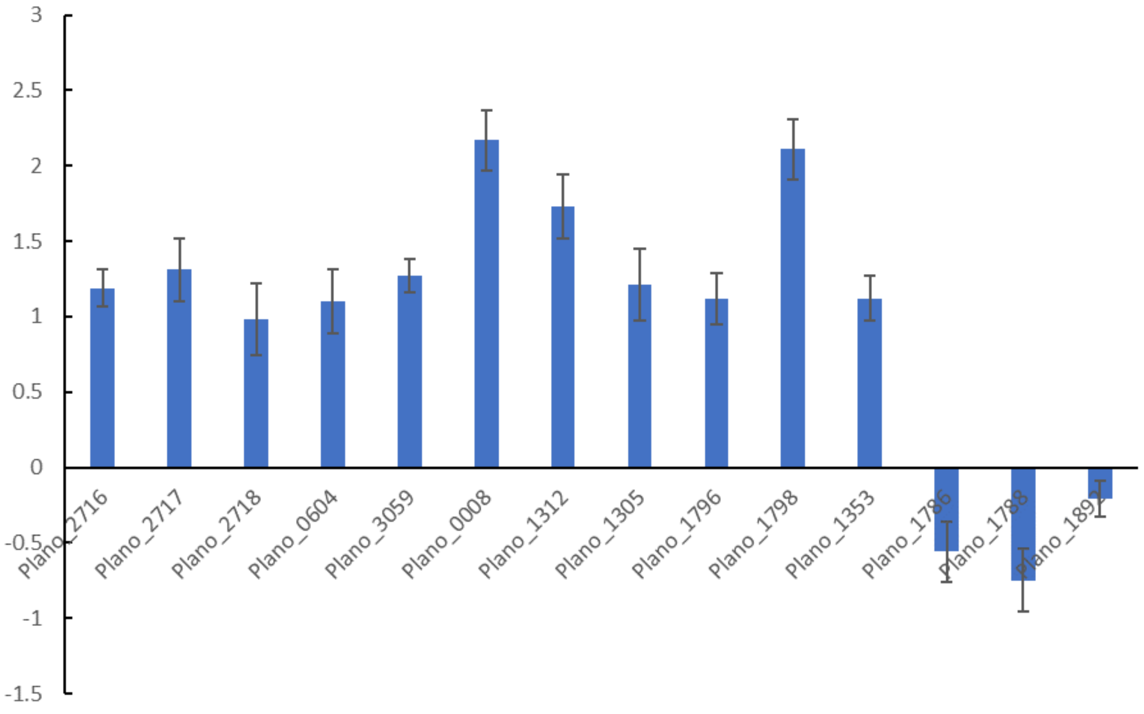

Fig. S1. RNA-seq data were validated by qRT-PCR. The blue bars show the expression levels of the DEGs in mutant strains R07 relative to wild type samples at 24 h.
